# Supplementary material for: A Critical View of the Application of the APEX Software (Aqueous Photochemistry of Environmentally-Occurring Xenobiotics) to Predict Photoreaction Kinetics in Surface Freshwaters
Source: Molecules. 2019 Dec 18;25(1):9. doi: 10.3390/molecules25010009 (PMC7017383; doi:10.3390/molecules25010009)
Supplement: Supplementary file 1 [file molecules-25-00009-s001.zip › 3.2.4_gcc-4.4.0.zip!/3.2.4_gcc-4.4.0/3.2.4_gcc-4.4.0/doc/octave/html/interpreter/EOF-and-Errors.html]

EOF and Errors - Untitled


Next: File Positioning,
Previous: Temporary Files,
Up: C-Style I/O Functions


---

#### 14.2.18 End of File and Errors

Once a file has been opened its status can be acquired. As an example
the `feof` functions determines if the end of the file has been
reached. This can be very useful when reading small parts of a file
at a time. The following example shows how to read one line at a time
from a file until the end has been reached.

```
     filename = "myfile.txt";
     fid = fopen (filename, "r");
     while (! feof (fid) )
       text_line = fgetl (fid);
     endwhile
     fclose (fid);
```

Note that in some situations it is more efficient to read the entire
contents of a file and then process it, than it is to read it line by
line. This has the potential advantage of removing the loop in the
above code.

— Built-in Function: **feof** (fid)  
> Return 1 if an end-of-file condition has been encountered for a given
> file and 0 otherwise. Note that it will only return 1 if the end of the
> file has already been encountered, not if the next read operation will
> result in an end-of-file condition.
>
>
>
> **See also:** fread, fopen, fclose.

— Built-in Function: **ferror** (fid)  
> Return 1 if an error condition has been encountered for a given file
> and 0 otherwise. Note that it will only return 1 if an error has
> already been encountered, not if the next operation will result in an
> error condition.

— Built-in Function: **fclear** (fid)  
> Clear the stream state for the specified file.

— Built-in Function: **freport** ()  
> Print a list of which files have been opened, and whether they are open
> for reading, writing, or both. For example,
>
> ```
>           freport ()
>           
>                -|  number  mode  name
>                -|
>                -|       0     r  stdin
>                -|       1     w  stdout
>                -|       2     w  stderr
>                -|       3     r  myfile
> ```
